# Molecular and Clinical Significance of Stanniocalcin-1 Expression in Breast Cancer Through Promotion of Homologous Recombination-Mediated DNA Damage Repair

**DOI:** 10.3389/fcell.2021.731086

**Published:** 2021-10-15

**Authors:** Jing Hou, Jigan Cheng, ZeHua Dai, Na Wei, Huan Chen, Shu Wang, Min Dai, Leilei Li, Hua Wang, Qing Ni

**Affiliations:** ^1^Department of Breast Surgery, Guizhou Provincial People’s Hospital, Guiyang, China; ^2^Department of Clinical Medicine, Guizhou Medical University, Guiyang, China

**Keywords:** breast cancer, stanniocalcin-1, homologous recombination, BRCA1, survival

## Abstract

Stanniocalcin-1 (STC1) is a glycoprotein hormone whose abnormal expression has been reported to be associated with a variety of tumors, but its function in breast cancer is not well understood. Through modulation of STC1 expression in different breast cancer cell lines, our study found that STC1 could promote the proliferation and growth of breast cancer cells and promote metastasis. Furthermore, STC1 reduced apoptosis induction by irradiation. We also found that STC1 could promote a homologous recombination-mediated DNA damage repair by recruiting BRCA1 to sites of damage. Moreover, STC1 silencing sensitized breast cancer cells to treatment with irradiation (IR), olaparib, or cisplatin *in vitro*. In clinical settings, the serum concentration of STC1 was higher in breast cancer patients than in healthy women, as detected by enzyme-linked immunosorbent assay (ELISA). In addition, immunohistochemical staining of breast cancer specimens showed that a high expression of STC1 was negatively correlated with recurrence-free survival in breast cancer, indicating that STC1 expression could be used as a predictive marker for a poor prognosis in breast cancer. All these findings indicate that STC1 promotes breast cancer tumorigenesis and that breast cancers with a high level of STC1 are more resistant to treatment, probably through homologous recombination (HR) promotion. Furthermore, combining STC1 inhibition and DNA damage-inducing drugs may be a novel approach to improve the survival of patients with STC1-expressing breast cancer.

## Introduction

Stanniocalcin (STC) is a member of the secretory glycoprotein family. It was first found in fish and, in recent years, has been widely found in humans and mammals. In mammals, STC mainly participates in the regulation of multiple physiological functions through autocrine or paracrine mechanisms, such as the regulation of the calcium and phosphorus balance in the body (Yeung et al., 2012; Zhao et al., 2020). The STC family is composed of STC1 and STC2. The STC1 gene was identified in 1995 and is located on chromosome 8p11.2-p21. STC1 can participate in the processes of oxidative stress, apoptosis, and inflammation. It has been reported that STC1 is associated with a variety of tumors, such as rectal cancer, hepatocellular carcinoma, medullary thyroid carcinoma, acute leukemia, breast cancer, and ovarian cancer (Kanellis et al., 2004; Nguyen et al., 2009; Sheikh-Hamad, 2010). However, many studies on the relationship between STC1 and tumor development have reported contradictory conclusions. Some studies have shown that STC1 is an oncogene, while others have shown that STC1 has an anticancer effect, indicating heterogeneous effects mediated by STC1 (Liu et al., 2010; Guo et al., 2013). Research on STC1 is still attractive because STC1 can be easily detected in the blood and is potentially a predictive marker for tumor diagnosis and treatment. Our previous study (Hou et al., 2015) showed that STC2 had little effect on the proliferation of breast cancer cells but could inhibit the invasion and metastasis of breast cancer cells. However, the function of STC1 in breast cancer is still elusive. Some studies have shown that the expression of STC1 is related to estrogen receptor (ER) and that STC1 is an estrogen-responsive gene (Bouras et al., 2002). Others have shown that the expression of STC1 can be regulated by BRCA1 at the transcriptional level (Welcsh et al., 2002). Recently, Han et al. (2016) found that STC1 is a prognostic indicator of triple-negative breast cancer (TNBC). Compared with non-TNBC cancers, the expression of STC1 in TNBC is higher, and the survival of TNBC patients with a high expression of STC1 is relatively poor. Since TNBC has been reported to harbor a high probability of BRCA1 deficiency and a homologous recombination defect (HRD), we hypothesized that STC1 has an important role in DNA repair and can be used as a biomarker for DNA damage-inducing agents in breast cancer.

## Materials and Methods

### Cell Lines and Cell Culture

The MCF-7, ZR-7530, MDA-MB-231 (231), and U2OS cell lines and lentiviral packaging cells (293T cells) were purchased from the American Type Culture Collection (ATCC). The MDA-MB-231 HM (231 HM) cell line was a gift from the Breast Cancer Institute of the Fudan University Shanghai Cancer Center. All cell lines were cultured in DMEM supplemented with 10% fetal bovine serum, penicillin (100 units/ml), and streptomycin (100 μg/ml). All cell cultures were incubated at 37°C in a 5% CO_2_ atmosphere. Mycoplasma contamination was monitored regularly by using Hoechst 33258 staining.

### Chemicals

Cisplatin and olaparib were purchased from Selleck. Cisplatin was dissolved in water at a stock concentration of 2 mM, aliquoted into 100-μl aliquots, and stored in a −80°C freezer. Each stock vial was used only once. Olaparib was dissolved in DMSO at a stock concentration of 200 mM, aliquoted into 20-μl aliquots, and stored in a −80°C freezer. Each stock vial was used only once. The final DMSO concentration of the solutions used in the study did not exceed 0.1%.

### Plasmids

pCMV6-STC1 was purchased from Origene (#RC206573). Full-length STC1 was inserted into the pLVX3 lentiviral vector by using the *Bam*HI and *Eco*RI sites. The forward primer was 5′- ACTGGATCCATGCTCCAAAACTCAGCAGT -3′, and the reverse primer was 5′- ACGTGAATTCTTATGCACTCTCATGGGAT -3′. The sequence of the STC1 insert was verified by DNA sequencing. STC1-specific shRNA was purchased from Sigma (#TRCN0000413339 and #TRCN0000151758). Lentiviruses expressing STC1 cDNA or STC1-specific shRNA and their corresponding empty vectors were packaged by transfection of plasmids into 293T cells. Virus-infected target cells were selected with puromycin (1 μg/ml) for 10–14 days to establish stable cell lines.

### Cell Proliferation and Viability Assays

Approximately 2 × 10^3^ cells were incubated in 96-well plates in 100 μl medium. The cells were cultured for 12 h, after which a basal time point measurement was recorded. Then, the cells were cultured for 1, 2, 3, 4, or 5 days and evaluated using Cell Counting Kit-8 (CCK-8). The absorbance at 450 nm was measured using a microplate reader. For the cell viability assay, cells were seeded at 2–4 × 10^3^ cells per well in 96-well plates in 100 μl medium, and then, DNA damage-inducing drugs were added at the indicated concentrations for 3 days. An MTS solution (1/10, *v*/*v*) was added to each well, and the cells were incubated for 2–4 h, depending on the cell type. The absorbance of each well at 490 nm was measured by subtracting the background (without cells) absorbance measurement. The mean value was calculated as the average of triplicates, and then, the percentage was calculated as the value of drug-treated cells minus that of time-matched vehicle-treated cells.

### Migration Assays

For the cell migration assay, a high-throughput screening multiwell-insert 24-well two-chamber plate with an 8-μm (pore size) polycarbonate filter between the chambers was used. MCF-7 STC1, ZR-7530 STC1, or MDA-MD-231 shSTC1 and MDA-MD-231 HM shSTC1 cells (4 × 10^4^) and their corresponding controls were added to the upper chamber and allowed to migrate at 37°C for 6–12 h toward the lower reservoir containing a medium supplemented with 2.5% fetal bovine serum. The migrated cells were fixed with ice-cold methanol for 30 min and stained with 0.1% crystal violet for 30 min. The non-migrated cells on the upper side of the insert membrane were removed. Then, the cells were imaged and counted at × 200 magnification under a microscope. Experiments were performed in triplicate.

### Cell Apoptosis Assay

To detect apoptosis, 1 × 10^5^ cells were given the indicated treatment and then stained with Annexin V and propidium iodide according to the protocol of the Annexin V-FITC Apoptosis Detection Kit (BD Biosciences, NJ, United States, catalog number: 556547); the stained cells were then subjected to flow cytometric analysis (Thermo Fisher, Attune NxT Flow Cytometer). The percentage of apoptotic cells was calculated. The experiment was performed in triplicate and repeated three times.

### Real-Time PCR

Total RNA was isolated from 2 × 10^6^ cells by using the Qiagen RNeasy Mini Kit. cDNAs were generated by reverse transcription and used for real-time PCR analysis with an ExScript RT-PCR kit (TaKaRa, Japan). The qPCR primers for STC1 were as follows: forward, 5′- AGCTGCCCAATCACTTCTCC -3′ and reverse, 5′- CTCATTGGTGCGTCTCCTGT -3′. The qPCR primers for GAPDH were as follows: forward, 5′- ACCCAGAA GACTGTGGATGG -3′ and reverse, 5′-TCTAGACGGCAGGTC AGGTC -3′. All amplifications and detections were carried out on a LightCycler 480 system (Roche, Basel, Switzerland). Statistical analyses were performed using the 2^–△△*CT*^ relative quantification method.

### Homologous Recombination and Non-homologous End-Joining Reporter Assay

The homologous recombination (HR) and non-homologous end-joining (NHEJ) reporter assays were performed according to the protocol reported previously in the literature (Gunn and Stark, 2012; Cavallo et al., 2021). DR-GFP and EJ5-GFP reporter systems were established to check repair efficiency, and plasmids (DR-GFP, EJ5-GFP, pCBA-I-SceI, and mCherry) were gifts from Professor Zhenkun Lou (Mayo Clinic). Briefly, target cells were seeded in 24-well plates. The cells were then transfected by Mirus reagent, with 100 ng of HR reporter (DR-GFP) or 100 ng of NHEJ reporter (EJ5-GFP) along with 100 ng of pCBA-I-SceI and 50 ng of mCherry at a ratio of 2:2:1 after 24 h. Forty-eight h later, the cells were collected and subjected to flow cytometric analysis (Thermo Fisher, Attune NxT Flow Cytometer). The results were analyzed by flow cytometric analysis (FACS) for RFP-positive cells and RFP/GFP both-positive cells. The relative percentage of GFP-positive cells was calculated by dividing the number of RFP/GFP both-positive cells with RFP single-positive cells.

### Western Blotting and Immunoprecipitation

After receiving the indicated treatment, cells were lysed with NETN buffer [20 mM Tris-HCl (pH 8.0), 0.1 mM EDTA, 100 mM NaCl, 0.5% NP-40 with 10 mM NaF, and protease inhibitors] in a dish on ice for 30 min before centrifugation. Then, the supernatants were immunoprecipitated with 20 μl Flag agarose beads (Sigma) overnight at 4 °C. The immunoprecipitates were rinsed using NETN three times and then boiled with 50 μl 2x Laemmli buffer. Immunoblotting was performed to detect the interaction of BRCA1 and STC1. An anti-BRCA1 antibody (sc-6954, 1:500 for western blotting) was purchased from Santa Cruz. Antibodies against STC1 (ab239518, 1:1,000 for western blotting), GAPDH (ab8245, 1:3,000 for western blotting), and β-actin (ab8226, 1:3,000 for western blot) were purchased from Abcam. An anti-Flag antibody (F3165, 1:3,000 for western blotting) was purchased from Millipore.

### Colony Formation Assay

Cells (500–800) were seeded in six-well plates in triplicate and treated with different drugs at the indicated concentration or IR after 24 h. Then, the cells were incubated for 12–14 days, fixed with methanol (30 min, RT), stained with 0.1% Giemsa (30 min, RT), and counted.

### Immunofluorescence Staining

Immunofluorescence staining was carried out following a standard process. Briefly, cells were plated on coverslips. After receiving the indicated treatments, cells were fixed in 4% paraformaldehyde for 20 min at RT, permeabilized in 0.5% Triton-X (5 min, RT), and blocked with 5% BSA in PBS. After washing with PBS three times, the cells were incubated with a primary antibody (overnight, 4°C). Next, the cells were washed and incubated with secondary antibodies (20 min, 37°C). DAPI (1 μg/ml) was used to counterstain nuclei. The cells were fixed with an anti-fade agent [p-phenylenediamine (0.1 mg/ml) dissolved in 90% glycerol in PBS]. Foci were imaged and counted using an ImageXpress confocal high-content imaging system (Molecular Device).

### Plasma Stanniocalcin-1 Measurement

Blood samples were drawn in the early morning after overnight fasting, and approximately 3 ml of venous blood was collected. The tubes used for collecting blood were common yellow-headed coagulant tubes in hospitals, which contained a separation gel and coagulant. The collected samples were stored in a refrigerator (BCD-450WDSD, Wuhan Province, China) at 4°C for 2 h and then centrifuged at 2,000 × *g* for 10 min. The supernatants were collected in a sterile environment and stored in an ultralow-temperature freezer (TSU700V, Massachusetts State, United States) at −80°C for subsequent experiments. The concentrations of plasma STC1 were determined using commercial enzyme immunoassay kits (ZCIBIO, Katy, United States). The intraassay coefficient of variation was < 10% for STC1. Enzyme-linked immunosorbent assay (ELISA) analysis was carefully performed according to the instructions of the manufacturer, and the assay has excellent specificity for detecting human STC1 with no significant cross-reactivity or observed interference with analogs according to the manufacturer. Individual samples were measured in triplicate in a single experiment.

### Immunohistochemistry

The expression of STC1 was analyzed using a tissue microarray (TMA) from Shanghai Outdo Biotech Company (China). The TMA contained 137 primary breast cancer tissues and 65 adjacent normal breast tissues. All the samples were from patients who underwent surgery. None of the patients received prior chemotherapy or radiotherapy. Clinical and demographic information, including age, sex, tumor localization, clinical staging, differentiation grade, receptor status, tumor size, and survival from the time of diagnosis, were available with patient consent. Samples were processed using routine methods for immunohistochemistry (IHC). ImageJ was used for image analysis. Three independent pathologists blinded to sample identity scored all of the samples. The final intensity score was determined by majority vote. Each specimen was scored according to staining intensity (no staining, 0; slight staining, (1) moderate staining, (2) and strong staining, (3) and staining area percentage (negative, 0; < 10% positive cells, 1; 11–50% positive cells, 2; 51–80% positive cells, 3; and > 80% positive cells, (4) The specimens were divided into two groups according to the product of the staining intensity and staining area percentage scores, where a product ≤ 1 was defined as the low-expression group and a product > 1 was defined as the high-expression group. Then, a survival curve was constructed with the survival times of the two groups.

### Statistics

All the data are represented as the mean ± SEM. To compare differences between experimental groups, two-way Student’s *t*-test was adopted. Statistical significance is represented as follows: ^∗^*P* < 0.05; ^∗∗^*P* < 0.01, ^∗∗∗^*P* < 0.001; and N.S. (not significant). *P* < 0.05 was considered to indicate a significant difference.

## Results

### Stanniocalcin-1 Promotes Breast Cancer Cell Proliferation

**FIGURE 1:**
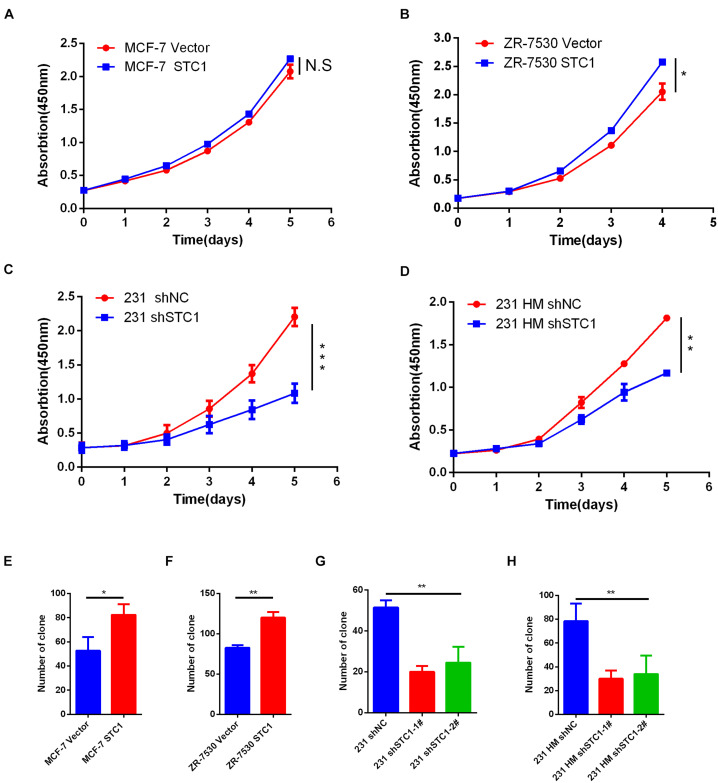
STC1 promotes breast cancer cell proliferation. **(A–D)** Detection of cell proliferation by Cell Counting Kit-8 (CCK8). Breast cancer cell lines transduced with STC1 cDNA (MCF-7 or ZR-7530) or shRNA [MDA-MB-231 (231) or MDA-MB-231 HM (231 HM)] were incubated in 96-well culture plates for 12 h to allow for attachment; after which, a 0-time point measurement was determined. After culturing for 1, 2, 3, 4, or 5 days, cell growth was detected using CCK-8. Absorbance at 450 nm was measured using a microplate reader. **(E–H)** Colony formation assay. MCF-7 STC1 and ZR-7530 STC1 cells or 231 shSTC1 and 231 HM shSTC1 cells and their corresponding control cells were seeded in six-well plates, and the cells were incubated for 12–14 days, fixed with methanol (30 min, RT), stained with 0.1% Giemsa (30 min, RT), and quantified. Data are shown as mean ± SEM from three independent experiments. *P*-value was determined by two-tailed unpaired *t*-test (*P < 0.05; **P < 0.01; ***P < 0.001; N.S., no significance).

To investigate the function of STC1 in breast cancer cells, we first evaluated the expression of STC1 in different breast cancer cell lines and found that the expression of STC1 in MCF-7 and ZR-7530 cells was low, while the expression in MDA-MB-231 (231) and MDA-MB-231 HM (231 HM) cells was relatively high (Supplementary Figures 1A,B). Therefore, we chose the breast cancer cell lines MCF-7 and ZR7530 for experiments on the overexpression of STC1 and the 231 and 231 HM cell lines for STC1-silencing experiments. Establishment of cell lines was verified by qPCR and western blotting (Supplementary Figure 1). A cell proliferation assay showed that STC1 overexpression promoted the proliferation of ZR-7530 cells but had little effect on MCF-7 cells (Figures 1A,B). Correspondingly, silencing STC1 significantly inhibited the growth of 231 and 231 HM cells (Figures 1C,D). Furthermore, a colony formation assay demonstrated similar results: STC1 overexpression promoted the clonal growth of MCF-7 and ZR-7530 cells, while STC1 silencing significantly dampened the growth of 231 and 231 HM cells (Figures 1E–H).

### Stanniocalcin-1 Influences the Migration of Breast Cancer Cells

**FIGURE 2:**
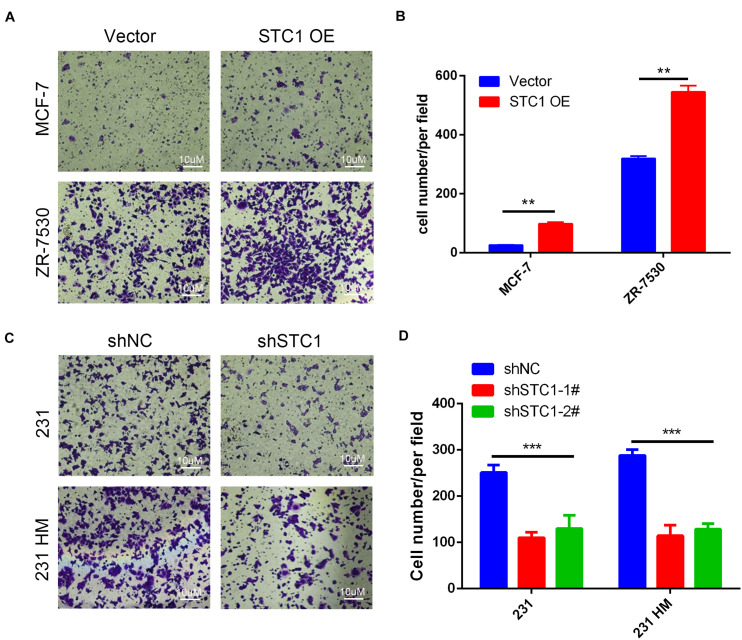
Stc1 influences the migration of breast cancer cells. **(A–D)** Migration assays. MCF-7 STC1 or ZR-7530 STC1 cells or 231 shSTC1 or 231 HM shSTC1 cells and their corresponding control cells were added in each upper chamber of a high-throughput screening multi-well insert 24-well two-chamber plate and allowed to migrate at 37°C for 6–12 h toward a lower reservoir containing a medium plus 2.5% fetal bovine serum. The invaded cells were fixed with ice-cold methanol for 30 min, stained with 0.1% crystal violet for 15 min, photographed, and counted at × 200 magnification under a microscope. **(A,C)** Representative images of migrated cells; **(B,D)** quantitative analysis of the number of migrated cells. Data are shown as mean ± SEM from three independent experiments. *P*-value was determined by two-tailed unpaired *t*-test (**P < 0.01; ***P < 0.001).

To investigate the effects of STC1 on the migration of breast cancer cells, we performed a migration assay using Transwell chamber plates. We found that more MCF-7 STC1 and ZR-7530 STC1 cells than corresponding control cells migrated through the membrane in the migration chamber (Figures 2A,B), but fewer 231 shSTC1 and 231 HM shSTC1 cells than corresponding control cells migrated through the membrane (Figures 2C,D).

### Stanniocalcin-1 Decreases Cell Apoptosis After Irradiation

**FIGURE 3:**
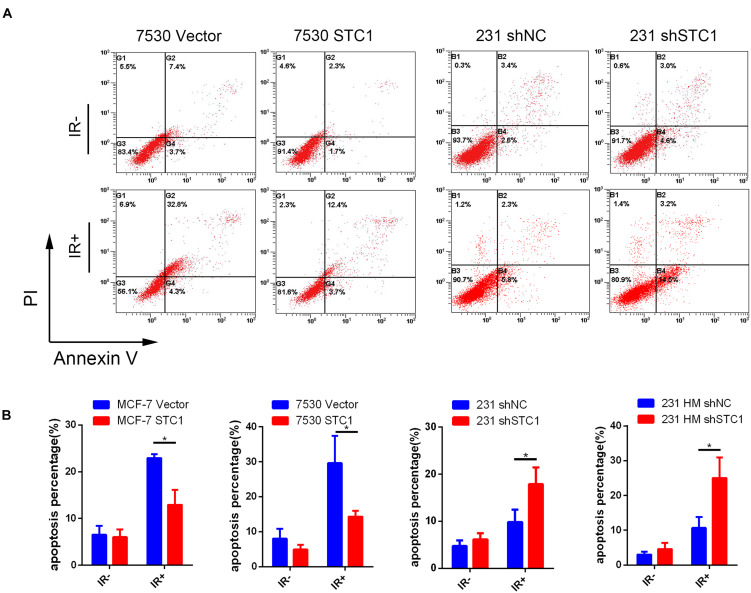
STC1 decreases cell apoptosis after irradiation. Cells were treated with a dose of 8-Gy X-rays, and apoptosis was then measured by flow cytometry in MCF-7 STC1, ZR-7530 STC1, or 231 shSTC1 cells and their corresponding control cells. **(A)** Representative images of apoptosis assay. **(B)** The percentage of apoptotic cells. Data are shown as mean ± SEM. *P*-value was determined by two-tailed unpaired *t*-test (*P < 0.05). IR +, with radiation treatment; IR−, without radiation treatment.

To explore the function of STC1 in breast cancer cells, we investigated the effects of STC1 on apoptosis in breast cancer cells by using a fluorescence-based Annexin V apoptosis assay. Because the baseline apoptosis of cancer cells was low, cells were treated with 8-Gy X-ray irradiation (IR), and the percentage of apoptotic cells was analyzed by FCM 24 h later. We found that the percentages of apoptotic MCF-7 STC1 and ZR7530 STC1 cells were lower than those of apoptotic control cells after irradiation, while silencing STC1 in 231 and 231 HM cells resulted in more apoptosis than that observed for control cells (Figure 3).

### Stanniocalcin-1 Enhances DNA Damage Repair in Breast Cancer Cells

**FIGURE 4:**
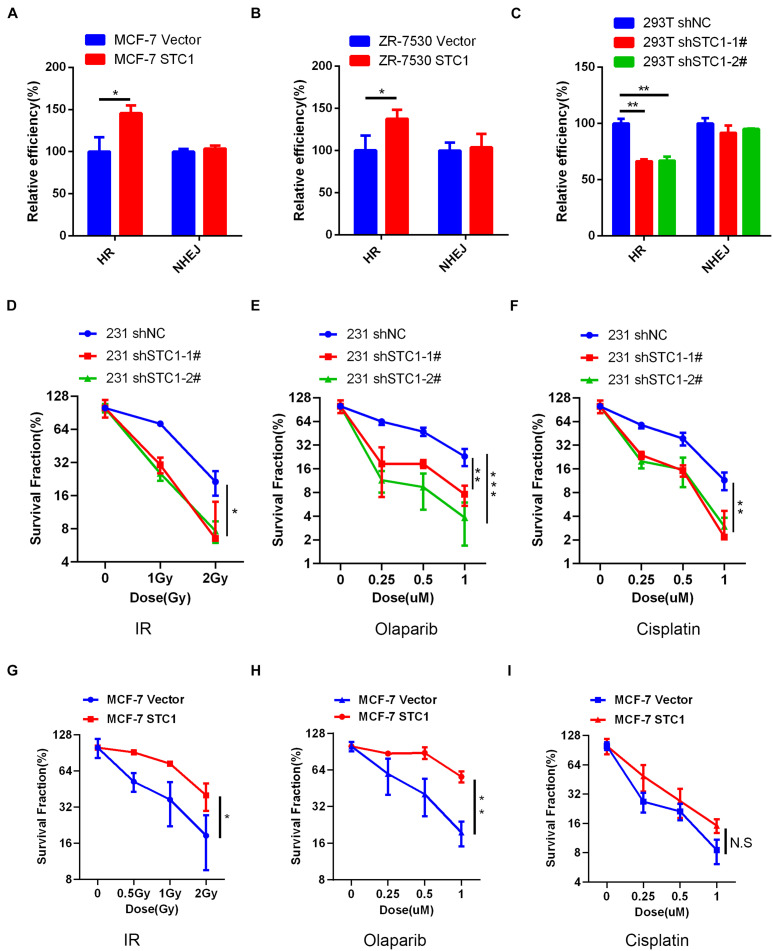
STC1 enhances DNA damage repair in breast cancer cells. **(A,B)** The homologous recombination (HR)/non-homologous end-joining (NHEJ) efficiency of MCF-7 cells **(A)** and ZR-7530 cells **(B)** overexpressed with STC1 or 293T cells with silencing of STC1 **(C)** was analyzed using HR/NHEJ reporter, respectively. **(D–F)** Colony formation analysis of 231 shSTC1 cells and the control cells treated with different doses of irradiation (IR), olaparib, or cisplatin. The survival fractions were normalized to 100% with no treatment for shNC. **(G–I)** Colony formation analysis of MCF-7 STC1 cells and the control cells treated with different doses of IR, olaparib, or cisplatin. The survival fractions were normalized to 100% with no treatment for MCF-7 vector. Data are shown as mean ± SEM from three independent experiments. *P*-value was determined by two-tailed unpaired *t*-test (*P < 0.05; **P < 0.01; ***P < 0.001).

To test whether STC1 can promote DNA repair after damage, we first employed a well-established DR-GFP reporter system to detect HR and NHEJ efficiencies after STC1 overexpression or silencing, as HR and NHEJ play important roles in DNA damage repair. As shown in Figures 4A–C, the overexpression of STC1 increased the HR efficiency of MCF-7 and ZR-7530 cells but had little effect on NHEJ, while silencing STC1 decreased the HR efficiency in 293T cells without an obvious influence on NHEJ. These results suggested that STC1 can facilitate DNA repair after damage. To further confirm this, we performed a colony formation assay to evaluate whether STC1 can modulate sensitivity to irradiation or DNA damage-inducing drugs such as olaparib and cisplatin. The results showed that loss of STC1 expression significantly sensitized 231 cells to irradiation, olaparib, and cisplatin (Figures 4D–F), while the overexpression of STC1 in MCF-7 cells rendered these cells more resistant to irradiation and olaparib. However, the overexpression of STC1 in MCF-7 cells had little effect on the sensitivity to cisplatin (Figures 4G–I). The above observations indicated an important role for STC1 in DNA damage repair, probably through regulation of HR.

### Stanniocalcin-1 Facilitates Homologous Recombination in a BRCA1-Mediated Manner

**FIGURE 5:**
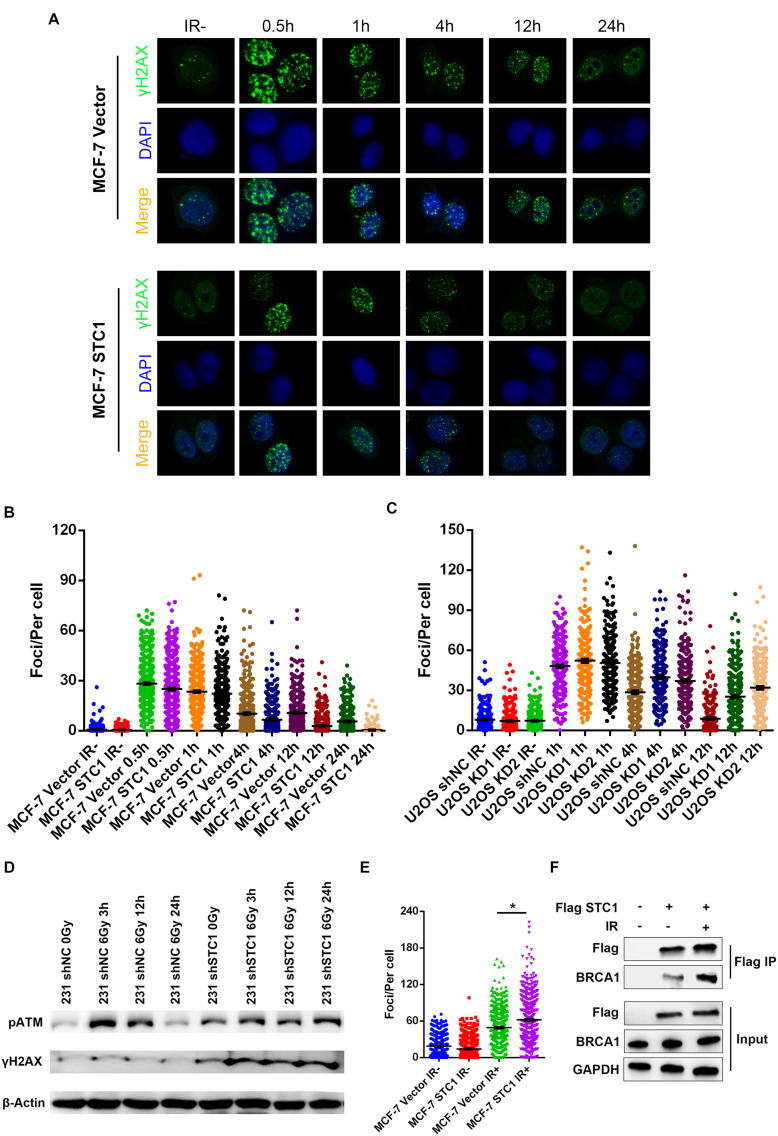
STC1 facilitates homologous recombination in a BRCA1-mediated manner. Representative images **(A)** and quantification of γH2AX foci in MCF-7 STC1 cells and their corresponding control cells **(B)** or U2OS cells stably expressing STC1 shRNA and their control cells **(C)**. Cells were treated with IR (2 Gy) at different time points and then were subjected to immunofluorescence with γH2AX antibody. The number of γH2AX was determined by ImageJ. **(D)** Immunoblot analysis of phosphorylation of ATM and H2AX in 231 shSTC1 cells and their control cells with or without IR. **(E)** Quantification of BRCA1 foci in MCF-7 STC1 cells and their corresponding control cells treated with IR (2 Gy) at 4-h time point. **(F)** The 293T cells were transfected with Flag-STC1 or vector and then treated with IR (10 Gy). One hour later, the cells were lysed and immunoprecipitated with anti-Flag agarose beads. The beads were boiled and blotted with indicated antibodies. *P*-value was determined by two-tailed unpaired *t* -test (*P < 0.05).

To explore the mechanism by which STC1 regulates HR, we performed immunofluorescence to detect γH2AX foci, a marker of DSBs, after irradiation. As shown in Figures 5A–C, STC1 overexpression decreased the number of γH2AX foci in MCF-7 cells at later time points after IR (4, 12, and 24 h); in contrast, silencing STC1 in U2OS cells increased γH2AX foci at these later time points. We also detected the phosphorylation of ATM and H2AX after IR and found that the phosphorylation of ATM and H2AX continued to be higher in 231 shSTC1 cells than in control cells at 24 h (Figure 5D), suggesting that loss of STC1 impairs DNA repair. Next, we wondered how STC1 is involved in the HR process. As it was previously reported that STC1 is related to BRCA1 in TNBC, we examined the effect of STC1 on the recruitment of the key HR component BRCA1 to DNA damage sites by detecting foci formation using an immunofluorescence assay. As shown in Figure 5E, STC1 overexpression led to a significant increase in BRCA1 foci upon IR treatment in MCF-7 cells, while silencing of STC1 in MDA-MB-468 cells decreased BRCA1 foci (Supplementary Figure 2B), indicating that STC1 facilitates the recruitment of BRCA1 to DNA damage sites. Besides, silencing of STC1 in MDA-MB-468 cells also decreased RAD51 foci (Supplementary Figure 2B). To further confirm this, we performed co-IP to assess the interaction between STC1 and BRCA1. The results showed that STC1 could interact with BRCA1 and that the interaction was increased after IR treatment (Figure 5F).

### High Expression of Stanniocalcin-1 Is Associated With an Advanced Disease Stage and Poor Survival in Breast Cancer Patients

**FIGURE 6:**
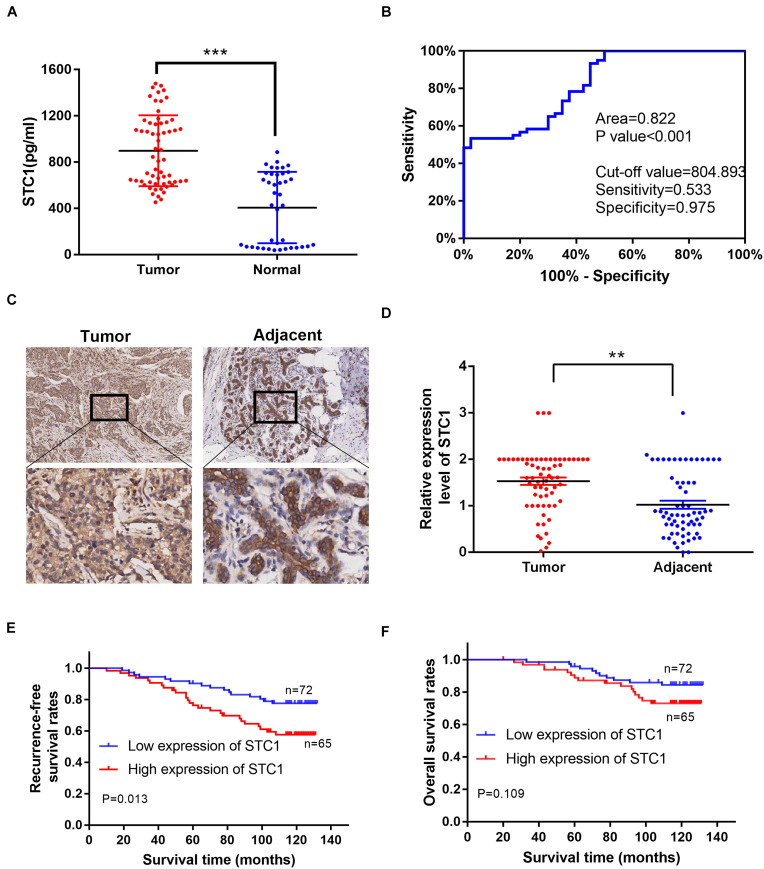
A high expression of STC1 is associated with an advanced disease stage and poor survival in breast cancer patients. **(A)** The concentration of STC1 protein in the serum of 60 breast cancer patients (tumor) and 40 healthy women (normal) detected by enzyme-linked immunosorbent assay (ELISA). **(B)** ROC curve analysis was used to determine the value of area under the curve (AUC). **(C)** Representative images of immunohistochemistry (IHC) staining of STC1 on tumor tissue or the adjacent area. **(D)** The relative expression of STC1 level between tumor tissue and the adjacent area measured by IHC. **(E,F)** Recurrence-free survival (RFS) and overall survival (OS) of breast cancer patients were analyzed by the Kaplan–Meier Plotter analysis. STC1 high: *n* = 65; LRRK2 low: *n* = 72. Data are shown as mean ± SEM. *P*-value was determined by two-tailed unpaired *t*-test (**P < 0.01; ***P < 0.001).

Our above data indicated that STC1 is important in the proliferation and migration of breast cancer cells, indicating that STC1 can promote tumorigenesis. STC1 also promotes HR and may lead to resistance to DNA damage-inducing drugs, thereby leading to poor survival in breast cancer patients. To determine the clinical significance of STC1 for breast cancer patients, we first measured the concentration of the STC1 protein in the serum of 60 breast cancer patients and 40 healthy women by ELISA, and the results showed that the concentration was higher in breast cancer patients than in healthy control women (Figure 6A). Furthermore, an ROC curve analysis was used to evaluate the diagnostic value of STC1. The area under the curve (AUC) was 0.822 (> 0.7), indicating a significant diagnostic value (*P* < 0.001) (Figure 6B). We also collected IHC data for 137 breast cancer patient samples and 65 adjacent normal breast tissue samples with detailed clinical information, including pathologic characteristics and survival information (Supplementary Table 1). The cutoff value of the cumulative score for STC1 immunostaining was defined as 1.0. Immunostaining for STC1 was designated positive if the score was ≥ 1.0. As shown in Figures 6C,D, the expression level of STC1 in breast cancer tissues was found to be significantly higher than that in adjacent tissues (*p* < 0.01). Notably, a high expression of STC1 was associated with worse recurrence-free survival (RFS) (Figure 6E). Although the overall survival (OS) of patients with a high expression of STC1 tended to be worse, this trend was not statistically significant (Figure 6F). In addition, a high expression of STC1 correlated with a more advanced clinical stage (Supplementary Table 1). Furthermore, a univariate analysis showed that STC1 expression (*p* = 0.015) was a predictor for OS. However, multivariate Cox analysis revealed that STC1 was not an independent prognostic factor for breast cancer (*p* > 0.05) (Supplementary Table 2). Collectively, our results suggest that STC1 promotes tumorigenesis in breast cancer and that breast cancer patients with a high expression of STC1 are more resistant to treatment, potentially through promotion of the HR pathway, and therefore have worse survival.

## Discussion

Research on STC1 is still attractive, as this protein can be easily detected in the serum and be used as a diagnostic or predictive marker for a subset of patients. However, the molecular function of STC1 is still elusive due to heterogeneity among different cancer types, leading to contradictory results and dampening the application potential of STC1 in clinical settings. Certainly, many studies have demonstrated that STC1 can regulate cell proliferation. For instance, STC1 was shown to inhibit the proliferation of cervical cancer cells (Guo et al., 2013) but promote tumor proliferation and cell colony formation in ovarian cancer, indicating that the effects of STC1 vary among different cancers. In our present study, we first confirmed the effects of STC1 on cell proliferation and migration and found that STC1 could promote breast cell proliferation and migration. Our results are consistent with those reported in Welcsh et al. (2002) and Daniel and Lange (2009), whose conclusions support that STC1 contributes to breast cancer cell proliferation. The potential mechanism underlying these observations may involve increased activity of cell cycle-regulated proteins and antiapoptotic proteins, as well as inhibition of the activity of caspase-3/caspase-9 (Liu et al., 2010). In line with these clues, our results showed that STC1 inhibited cell apoptosis after IR. It has been reported that *STC1* and *STC2* are estrogen-responsive genes; therefore, the effects of STC1 on cell proliferation may be related to the ER signaling pathway (Bouras et al., 2002). Interestingly, silencing STC1 in the ER-negative breast cancer cell line MDA-MB-231 also obviously dampened cell growth, suggesting the involvement of other mechanisms. In addition, it was observed that silencing STC1 expression reduced tumor growth in both murine and human breast cancer cells *in vivo*, but no obvious effects on proliferation were observed in either model *in vitro* (Chang et al., 2015), implying that the cell microenvironment is important for STC1 function. A recent paper reported that tumor STC1 inhibits APC phagocytosis and contributes to tumor immune evasion and immunotherapy resistance (Lin et al., 2021), indicating a role for STC1 in the immune response, which provides a possible reason to explain why STC1 functions differently *in vivo* and *in vitro*. Recent studies have suggested that STC1 is associated with invasion and metastasis, especially in TNBC. Murai et al. (2014) showed that STC1 enhanced cell invasion by the MDA-MB-231 cell line *in vitro* and promoted lung metastasis *in vivo*. Another study showed that STC1 silencing reduced cell metastasis by murine cell lines and the MDA-MB-231 cell line (Chang et al., 2015). Furthermore, similar results were observed in two other studies performed by Han et al. (2016) and Jeon et al. (2016), in which the overexpression of STC1 was found to significantly increase the invasiveness and metastasis of TNBC cells. These results are similar to our data showing that downregulation of STC1 reduced the metastatic capability of 231 cells and 231 HM cells. The detailed mechanism by which STC1 stimulates cell invasion and metastasis remains to be fully elucidated. In our study, we hypothesized that STC1 may function though DNA damage repair in a BRCA1-dependent manner based on the following facts: a. the expression of STC1 in breast cancer is reported (Welcsh et al., 2002) to be related to BRCA1; b. Han et al.(Han et al., 2016) demonstrated that high STC1 expression levels significantly increased the invasiveness of TNBC cells; c. approximately 20% of TNBC patients have a BRCA1 mutation (Gerratana et al., 2016), while 70% of patients with a BRCA1 mutation are diagnosed with TNBC (Comen et al., 2011); and d. our results showed that silencing of STC1 in 231 cells decreased cell apoptosis after IR compared to the apoptosis of control cells. In favor of our hypothesis, we provided evidence that STC1 could increase the HR efficiency induced by the I-secI enzyme by employing a well-established reporter system. Furthermore, knockdown of STC1 sensitized 231 cells to IR and DNA damage-inducing agents such as cisplatin and olaparib. A mechanistic study revealed that STC1 facilitated DNA repair after DNA damage, as the intensity of γH2AX foci decreased more quickly when STC1 was introduced into MCF-7 cells, while the opposite phenomenon was observed when STC1 was silenced. We also demonstrated that STC1 could interact with BRCA1 and that this interaction increased upon irradiation, suggesting that STC1 can recruit BRCA1 to DNA damage sites. To our knowledge, no study has reported the function of STC1 in DNA damage repair. However, the mechanism by which STC1 facilitates such processes needs a more detailed investigation. Based on our observations, we can easily conclude that STC1 can promote cell proliferation, metastasis, and apoptosis after stress; therefore, STC1 boosts breast cancer tumorigenesis. Additionally, STC1 facilitates DNA damage repair, which can lead to treatment resistance and shortened survival in patients. Therefore, STC1 could be an ideal marker for the diagnosis of breast cancer and the prediction of survival. Recently, a number of studies have indicated that the protein and mRNA expression levels of STC1 may be valuable prognostic markers in breast cancer (Chen et al., 2019). McCudden et al. (2004) reported that patients with breast cancer with a high level of STC1 had an increased incidence of lymph node involvement. Similarly, Wascher et al. (2003) demonstrated that STC1 transcriptional levels in the bone marrow and blood of breast cancer patients were associated with tumor size, lymph node status, and overall survival. In addition, the study highlighted the potential application of STC1 as a valuable marker for occult breast cancer cells in the bone marrow and blood of patients (Wascher et al., 2003). Furthermore, a retrospective study of 1,457 clinical samples found a significant association between a high STC1 expression and a poor clinical outcome (Chang et al., 2015). Our clinical data showed that the serum concentration of STC1 was higher in breast cancer patients than in healthy women, as detected by ELISA. IHC evaluation of breast cancer specimens demonstrated that a high expression of STC1 was negatively correlated with recurrence-free survival in breast cancer, indicating that STC1 expression could be used as a predictive marker for a poor prognosis in breast cancer.

In conclusion, our findings indicate that STC1 promotes breast cancer tumorigenesis and that breast cancers with high levels of STC1 are more resistant to treatment, potentially mediated through HR promotion. Furthermore, combined treatment with STC1 inhibition and DNA damage-inducing drugs may be a novel strategy to improve the outcome of patients with STC1-expressing breast cancer.

## Data Availability Statement

The original contributions presented in the study are included in the article/Supplementary Material, further inquiries can be directed to the corresponding author/s.

## Ethics Statement

The studies involving human participants were reviewed and approved by Ethics Committee of Guizhou Provincial People’s Hospital. The patients/participants provided their written informed consent to participate in this study.

## Author Contributions

JH, JC, and QN designed and performed the experiments, prepared the figures, and interpreted the data. JH, JC, and ZD drafted the “*Results*” section of the manuscript. SW, NW, and ZD verified the statistical analysis and revised the manuscript. JH, HW, and MD conceived and designed the study, interpreted the data, wrote and revised the manuscript, and obtained funding to support the research. All authors contributed to the article and approved the submitted version.

## Conflict of Interest

The authors declare that the research was conducted in the absence of any commercial or financial relationships that could be construed as a potential conflict of interest.

## Publisher’s Note

All claims expressed in this article are solely those of the authors and do not necessarily represent those of their affiliated organizations, or those of the publisher, the editors and the reviewers. Any product that may be evaluated in this article, or claim that may be made by its manufacturer, is not guaranteed or endorsed by the publisher.

## Supplementary Material

Supplementary Figure 1 The baseline expression of STC1 in breast cancer cell lines and verification of STC1 overexpression or silencing. **(A,B)** The baseline expression of STC1 in three breast cancer cell lines determined by qPCR and western blot. **(C–G)** Verification of the establishment of STC1 overexpression or silencing cells by qPCR and western blot. Data are shown as mean ± SEM. *P*-value was determined by two-tailed unpaired *t*-test (^∗∗^*P* < 0.01; ^∗∗∗^*P* < 0.001).

Supplementary Figure 2 **(A)** Representative images of MCF-7 cells for apoptosis assay. **(B)** Quantification of BRCA1 and RAD51 foci in MDA-MB-468 (468) shSTC1 cells and their corresponding control cells treated with IR (2 Gy) at 4-h time point.
