# Supplementary figures and images for: Molecular and Clinical Significance of Stanniocalcin-1 Expression in Breast Cancer Through Promotion of Homologous Recombination-Mediated DNA Damage Repair
Source: Front Cell Dev Biol. 2021 Oct 15;9:731086. doi: 10.3389/fcell.2021.731086 (PMC8554131; doi:10.3389/fcell.2021.731086)

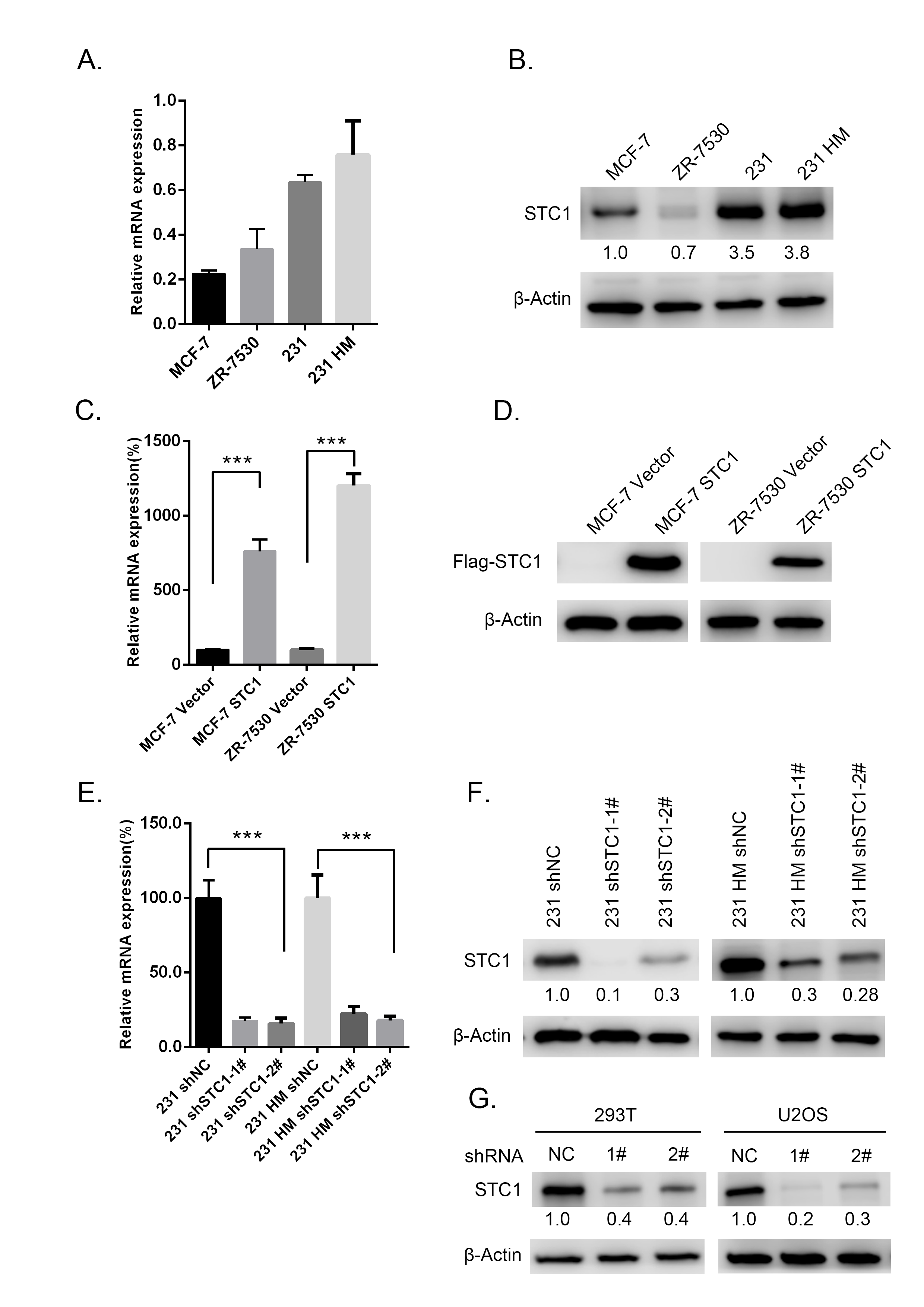

Supplement: Supplementary Figure 1 — The baseline expression of STC1 in breast cancer cell lines and verification of STC1 overexpression or silencing. (A,B) The baseline expression of STC1 in three breast cancer cell lines determined by qPCR and western blot. (C–G) Verification of the establishment of STC1 overexpression or silencing cells by qPCR and western blot. Data are shown as mean ± SEM. P-value was determined by two-tailed unpaired t-test (∗∗P < 0.01; ∗∗∗P < 0.001). [file Image_1.TIF]

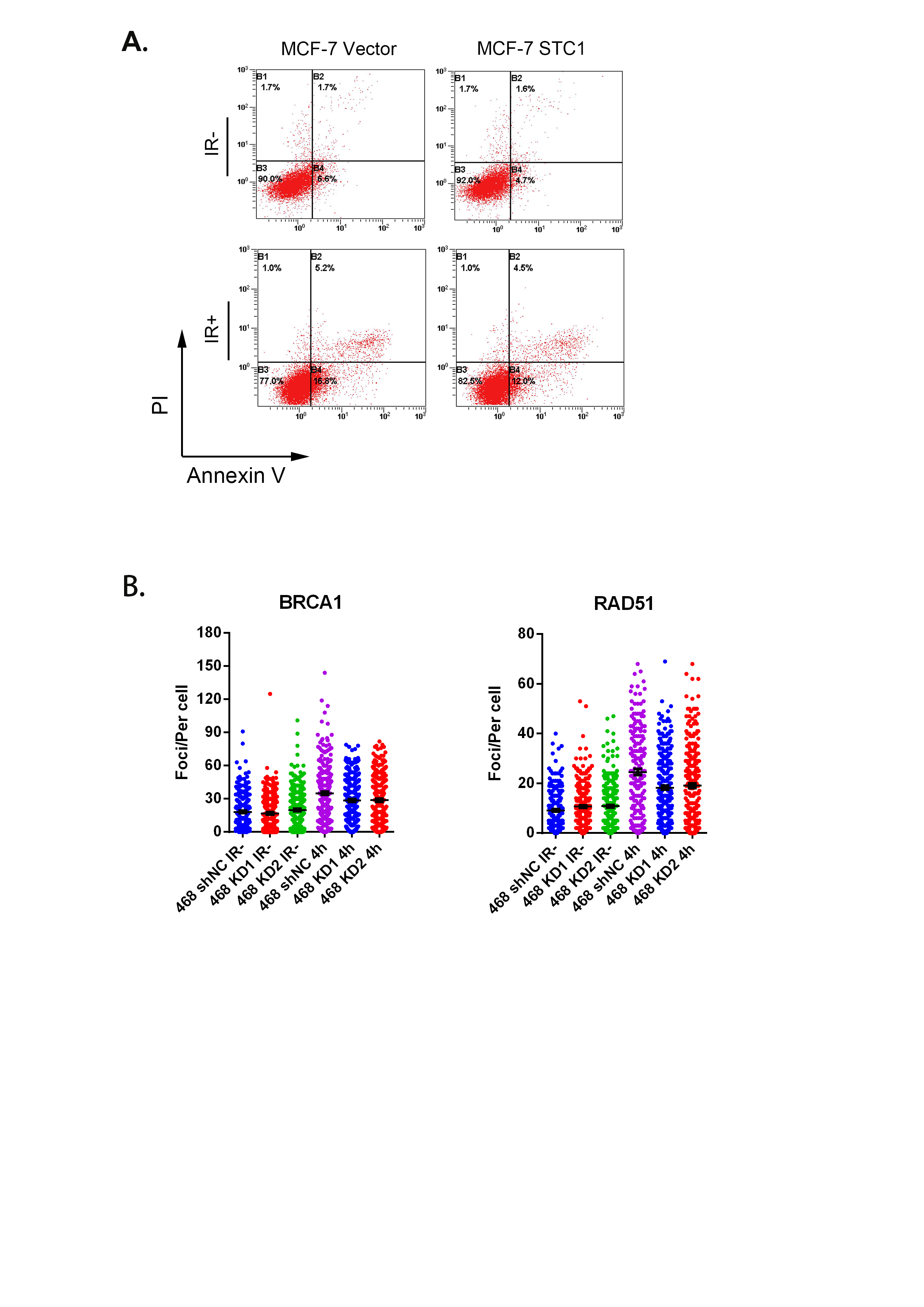

Supplement: Supplementary Figure 2 — (A) Representative images of MCF-7 cells for apoptosis assay. (B) Quantification of BRCA1 and RAD51 foci in MDA-MB-468 (468) shSTC1 cells and their corresponding control cells treated with IR (2 Gy) at 4-h time point. [file Image_2.TIF]
