# Supplementary material for: Molecular and Clinical Significance of Stanniocalcin-1 Expression in Breast Cancer Through Promotion of Homologous Recombination-Mediated DNA Damage Repair
Source: Front Cell Dev Biol. 2021 Oct 15;9:731086. doi: 10.3389/fcell.2021.731086 (PMC8554131; doi:10.3389/fcell.2021.731086)
Supplement: Supplementary file 3 [file Table_1.DOC]

**Supplementary table.1 Correlation between STC1 expression and clinicopathological characteristics**

|  | variables | **STC1 expression** | | total | χ2 | p value |
| --- | --- | --- | --- | --- | --- | --- |
|  |  | low | high |  |  |  |
| age |  |  |  |  | 1.636 | 0.201 |
|  | ≤50 | 40 | 29 | 69 |  |  |
|  | ＞50 | 32 | 36 | 68 |  |  |
| TNM stage |  |  |  |  | 6.014 | **0.049** |
|  | Ι | 20 | 12 | 32 |  |  |
|  | II | 35 | 25 | 60 |  |  |
|  | III | 17 | 28 | 45 |  |  |
| N stage |  |  |  |  | 3.077 | 0.079 |
|  | N0 | 44 | 30 | 74 |  |  |
|  | N1/N2/N3 | 28 | 35 | 63 |  |  |
| Her-2 |  |  |  |  | 0.056 | 0.813 |
|  | negative | 57 | 56 | 113 |  |  |
|  | positive | 13 | 11 | 24 |  |  |
| PR | negative |  |  |  | 8.788 | **0.003** |
|  | positive | 43 | 25 | 68 |  |  |
|  | negative | 28 | 41 | 69 |  |  |
| ER | positive |  |  |  | 0.258 | 0.611 |
|  | negative | 24 | 23 | 47 |  |  |
|  | positive | 46 | 44 | 90 |  |  |
| Lumina type |  |  |  |  | 1.809 | 0.613 |
|  | A | 37 | 37 | 74 |  |  |
|  | B | 7 | 9 | 16 |  |  |
|  | HER-2 | 14 | 8 | 22 |  |  |
|  | Triple negative | 14 | 11 | 25 |  |  |
| Pathologic type |  |  |  |  | 0.176 | 0.675 |
|  | invasive ductal carcinoma | 65 | 60 | 125 |  |  |
|  | other | 7 | 5 | 12 |  |  |
| grade |  |  |  |  | 3.597 | 0.058 |
|  | II | 68 | 55 | 123 |  |  |
|  | III | 4 | 10 | 14 |  |  |
| Vascular invasion |  |  |  |  | 0.563 | 0.453 |
|  | negative | 65 | 56 | 121 |  |  |
|  | positive | 7 | 9 | 16 |  |  |
