# Supplementary material for: Molecular and Clinical Significance of Stanniocalcin-1 Expression in Breast Cancer Through Promotion of Homologous Recombination-Mediated DNA Damage Repair
Source: Front Cell Dev Biol. 2021 Oct 15;9:731086. doi: 10.3389/fcell.2021.731086 (PMC8554131; doi:10.3389/fcell.2021.731086)
Supplement: Supplementary file 4 [file Table_2.DOC]

**Supplementary table.2 Univariate and multivariate analyses of** **prognostic factors for breast cancer**

| variables | Univariate analysis | | | |  | Multivariate analysis | | |  |
| --- | --- | --- | --- | --- | --- | --- | --- | --- | --- |
|  | | HR | 95%CI | p value |  | HR | 95%CI | p value |  |
| STC1 expression | | 2.17 | 1.163-4.05 | **0.015** |  | 1.604 | 0.846-3.038 | 0.147 |  |
| TNM | | 2.915 | 1.785-4.76 | **0** |  | 2.398 | 1.449-3.968 | **0.001** |  |
| Grade | | 5.327 | 2.658-10.678 | **0** |  | 3.074 | 1.464-6.453 | **0.003** |  |
| Age | | 1.473 | 0.799-2.715 | 0.214 |  |  |  |  |  |
| ER | | 0.454 | 0.248-0.832 | **0.011** |  | 0.666 | 0.35-1.267 | 0.215 |  |
| PR | | 0.943 | 0.515-1.728 | 0.85 |  |  |  |  |  |
| HER2 | | 1.661 | 0.827-3.337 | 0.154 |  |  |  |  |  |
